# Tea and coffee consumption, cognitive impairment and prognosis in older inhabitants

**DOI:** 10.17179/excli2020-2877

**Published:** 2020-10-02

**Authors:** Tomoyuki Kawada

**Affiliations:** 1 Department of Hygiene and Public Health, Nippon Medical School, 1-1-5 Sendagi, Bunkyo-Ku, Tokyo 113-8602, Japan

## ⁯

***Dear Editor,***

Several prospective studies have been conducted on the association between coffee/tea consumption, cognitive impairment and prognosis in older subjects. Here, I want to present the data and speculate on the inter-relationships.

Shirai et al. (2020[7]) reported that the adjusted hazard ratios (HRs) (95 % confidence intervals [CIs]) for cognitive decline among those who consumed green tea once/day, 2-3 times/day, and ≥ 4 times/day were 0.70 (0.45-1.06), 0.71 (0.52-0.97), and 0.72 (0.54-0.98), respectively. Although there was no dose-response relationship between green tea and cognitive decline, a reduction of about 30 % in cognitive decline was observed. In contrast, coffee intake had no significant effect on cognitive decline. Cornelis et al. (2020[2]) examined the associations of regular coffee or tea drinking and caffeine intake with cognitive function. They found that cognitive function significantly decreased with consumption of one or more cups of coffee or tea consumption. Panza et al. (2015[5]) conducted a systematic review and found that coffee, tea, and caffeine consumption or higher plasma caffeine levels were protective against cognitive impairment/decline and dementia. However, there was a lack of a distinct dose-response association, and the preventive effect was stronger among females than males. There was also a lack of a significant association in longer follow-up studies, and the association in late-life outcomes might have disappeared. Besides, the effect of tea and coffee intake on cognitive decline remains contentious.

Regarding prognosis, Shadyab et al. (2020[6]) conducted a follow-up study on older women aged 65 to 81 years at baseline. Caffeinated coffee, decaffeinated coffee, or caffeinated tea consumption was not significantly associated with survival to age 90 years after adjusting for confounders, regardless of smoking, body mass index, or race/ethnicity.

The levels of cognitive impairment give rise to an increased risk of mortality, which is apparent even for quite mild levels of impairment (Dewey and Saz, 2001[3]). In addition, co-occurring cognitive and physical limitations constitute a distinct risk in older people with mortality (Grande et al., 2020[4]). These reports suggest that lifestyle factors, such as coffee/tea consumption, might not directly contribute to subsequent mortality. Instead, physical disability and cognitive impairment might progress in combination with changes in daily life habits. Beydoun et al. (2014[1]) reported that there are significant associations between cognitive performance and modifiable dietary factors, such as caffeine intake, alcohol intakes, and overall nutrient adequacy score. Furthermore, they found stratum-specific associations by sex and baseline age between cognitive performance and modifiable lifestyle factors. A comprehensive survey is needed for the monitoring of sub-clinical changes in older people. 

## Conflict of interest

The author declares no conflict of interest.
